# Phytochemical and Pharmacological Properties of* Chaenomeles speciosa*: An Edible Medicinal Chinese Mugua

**DOI:** 10.1155/2018/9591845

**Published:** 2018-12-09

**Authors:** Weifeng Huang, Junwei He, Muhammad Farrukh Nisar, Hongshui Li, Chunpeng Wan

**Affiliations:** ^1^Department of Microbiology and Immunology, Medical College, China Three Gorges University, Yichang, Hubei 443002, China; ^2^Research Center of Natural Resources of Chinese Medicinal Materials and Ethnic Medicine, Jiangxi University of Traditional Chinese Medicine, Nanchang 330004, China; ^3^Interdisciplinary Research Centre in Biomedical Materials (IRCBM), COMSATS University Islamabad, Lahore Campus, Lahore 54000, Pakistan; ^4^The Second People Hospital of Dezhou, Dezhou 253022, China; ^5^Jiangxi Key Laboratory for Postharvest Technology and Nondestructive Testing of Fruits & Vegetables, Collaborative Innovation Center of Post-Harvest Key Technology and Quality Safety of Fruits and Vegetables, College of Agronomy, Jiangxi Agricultural University, Nanchang 330045, China

## Abstract

*Chaenomeles *plants are adapted to diverse ecological zones particularly the temperate areas of Korea, Japan, and China. In China,* Chaenomeles speciosa *is mainly planted in Chongqing, Anhui, and Hubei provinces. Most of the studies till date have been focused on the anti-inflammatory activities of* C. speciosa* fractions. The present study aimed to review the maximum literature reported for the presence of various phytochemicals in* C. speciosa*. In addition, the pharmacological properties of these chemical compounds of this plant shall also be discussed. The extracts of the various parts of the plant are rich in diversity of antioxidants, organic acids, phenolics, terpenoids, and many different phytochemicals that bear strong anticancer, antioxidant, antiviral, antibacterial properties, anti-inflammation, antihyperlipidemic, antihyperglycemic, and anti-Parkinson properties.* C. speciosa* fruits have broad scope in industry as well as in medicines. Not only the leaves and fruits of* C. speciosa* plant, but various other parts including roots, seeds, bark twigs, and flowers all have long history of clinical trials in curing many human ailments. However, the maximum accessible data concerning the chemical compositions and their broad pharmacological properties of* C. speciosa* plant parts is pretty restricted that make it more appealing for in-depth investigations.

## 1. Introduction

*Chaenomeles *plants are adapted to diverse ecological zones, but mostly occupied the temperate areas of Korea, Japan, and China. In China, it is mainly planted in Chongqing, Anhui, and Hubei provinces and locally called ‘Zhoupi mugua', which has been well documented in broad traditional Chinese herbal medication systems. Recent scientific studies unveil high nutritional value of this plant. In the past few decades, cultivation of* C. speciosa *became a part of routine agriculture and fulfilling an ever increasing demands of the industry particularly the fruits juices, fruits tea, vinegar and fruit preservation, etc. Many of the agriculture varieties have been introduced in the market, to increase the gross yield of which three traditional varieties are well known, i.e., Luohanji, Zimugua, and Changjun [1]. The extracts of the fruits of* C. speciosa* are rich in diverse antioxidants and many different phytochemicals that bear strong anticancer, antioxidant, antiviral, antibacterial properties, anti-inflammation, antihyperlipidemic, antihyperglycemic, and anti-Parkinson properties [2–6].

*Chaenomeles* are enriched with antioxidants and *α*-glucosidase inhibitory activities are well documented in recent studies [7]. Various parts of the* Chaenomeles* plant which have variable amounts of phytochemicals such as peels are rich in triterpenes, due to which it is highly antioxidant [7]. This is quiet common observation which is being supported by multiple studies, that peels of a variety of fruits such as pear and hawthorn are having more antioxidants [8]. In case of* Chaenomeles* most of its peels are being wasted and ultimately huge amount of good source of antioxidants is gone wasted [8]; hence* Chaenomeles* is a potential candidate in terms of superior performance due to its higher *α*-glucosidase and antioxidant functions [7]. In addition to these, various triterpenes and phenolics are also present in the plant extracts, especially ursolic and oleanolic acids which are potential chemicals being recognized even in People's Republic of China Pharmacopoeia. Moreover, various minor but active compounds,* namely*, chlorogenic acid, catechin, epicatechin, gallic acid, protocatechuic acid, caffeic acid, and syringic acid have also been isolated from* Chaenomeles* [9, 10].* C. speciosa *fruits are well studied for their ingredients and phytochemical compounds such as polyphenols and vitamin C [11], and studies depicted that it could be a good source for the natural antioxidants and sufficient amount of fibers and low amount of citric acid [11, 12].

*C. speciosa* is a member of family Rosaceae, and also known as flowering quince [13]. Traditionally,* C. speciosa* has widely been used in clinical trials to treat hepatitis, rheumatoid arthritis, and prosopalgia [14]. It is also used as an edible food, canned food, preserved fruit, fruit wine, fruit vinegar, and juices [13]. Most of the studies till date have been focused on the anti-inflammatory activities of* C. speciosa* fractions. The present study aimed to review the maximum literature reported for the presence of phytochemicals in* C. speciosa*. In addition, the pharmacological properties of these chemical compounds of this* Chaenomeles* fruits shall also be discussed.

## 2. Chemical Constituents

**Figure 1:**
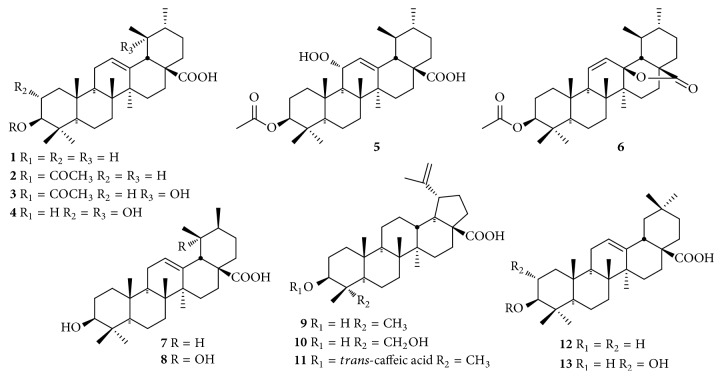
Chemical structures of triterpenes from* C. speciosa*.

**Figure 2:**
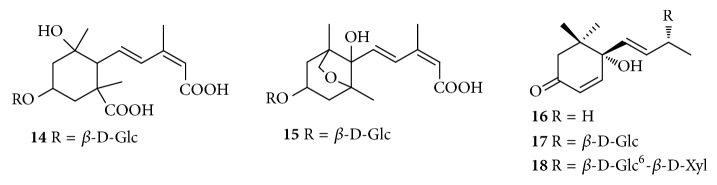
Chemical structures of sesquiterpenoids from* C. speciosa*.

**Figure 3:**
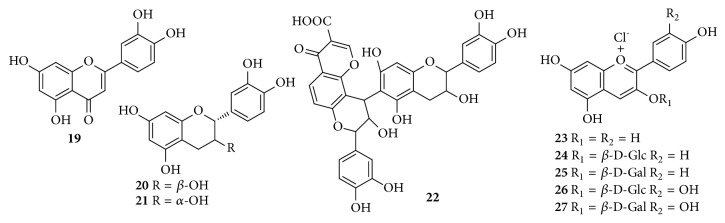
Chemical structures of flavonoids from* C. speciosa*.

**Figure 4:**
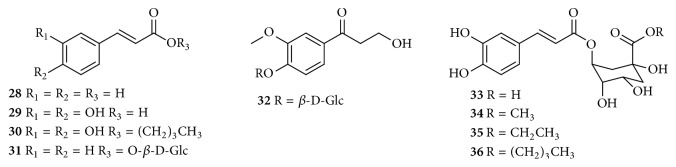
Chemical structures of phenylpropanoids from* C. speciosa*.

**Figure 5:**
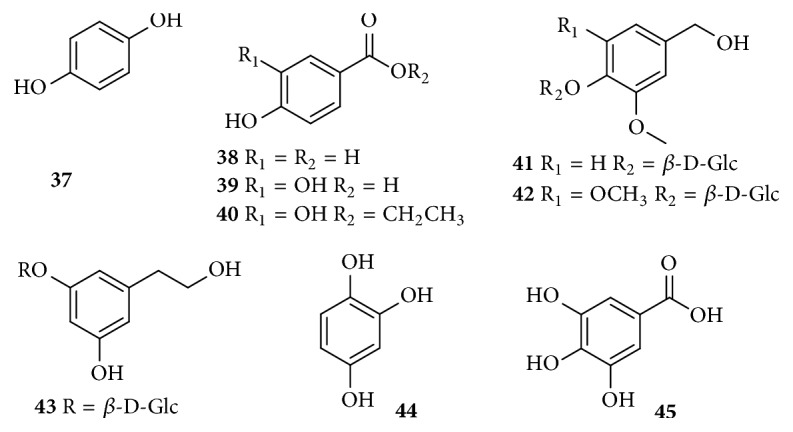
Chemical structures of phenolics from* C. speciosa*.

**Figure 6:**
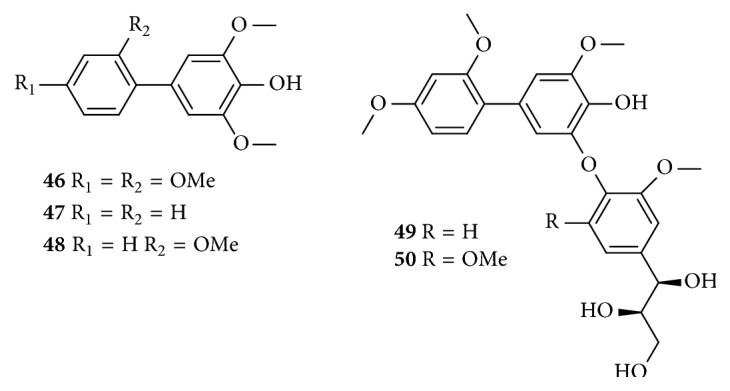
Chemical structures of biphenyls from* C. speciosa*.

**Figure 7:**
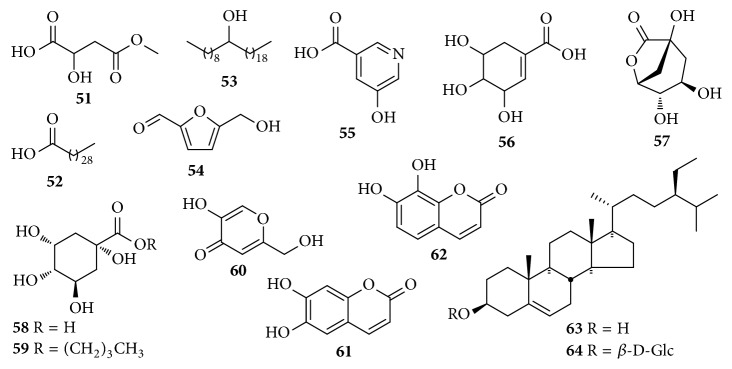
Chemical structures of other constituents from* C. speciosa*.

Up to now, approximately 64 chemical constituents have been isolated and identified from* C. speciosa*, including triterpenes, sesquiterpenoids, flavonoids, phenylpropanoids, phenols, biphenyls, and others. Among them, triterpenes and flavonoids were considered to be the primary bioactive constituents of* C. speciosa*. The components isolated from* C. speciosa* are summarized in the current review (Figures 1–7).

### 2.1. Triterpenes

Triterpenes are regarded as the major bioactive ingredients of* Chaenomeles* species, and phytochemical studies are focusing this genus since last two decades [15]. To date, 13 steroid compounds have been isolated and identified from* C. speciosa.* The steroids and theirs chemical structures are well described in recent studies (Table 1 and Figure 1). They all exist in the form of aglycones that belong to pentacyclic triterpenoids, including ursanes (**1-7**), lupanes (**8-11**), and oleananes (**12** and** 13**).

### 2.2. Sesquiterpenoids

Sesquiterpenoids represent a relatively small group of compounds in* Chaenomeles* species. To date, only five sesquiterpenoids were obtained from the ethanolic extract of* C*.* speciosa* so far. Their structures of these compounds are shown in Table 2 and Figure 2.

### 2.3. Flavonoids

Flavonoids are comprised of a huge number of polyphenolic compounds having a benzo-*γ*-pyrone organization, which is universally occurred in plant kingdom; there is no exception for* Chaenomeles* species. Flavonoids are the second major bioactive constituents in* Chaenomeles* species and they are divided into three categories including one flavone (**19**), three flavanonols (**20**–**22**), and five anthocyanins (**23**–**27**). Their structures of these compounds are shown in Table 3 and Figure 3.

### 2.4. Phenylpropanoids

*Chaenomeles* species are also rich in phenylpropanoids. Almost 9 phenylpropanoids were isolated and identified from* C. speciosa* to date. These phenylpropanoids include 8 phenylpropionic acids (**28–31** and** 33–36**) and one phenylpropanol (**32**). Their structures of these compounds are elaborated in Table 4 and Figure 4.

### 2.5. Phenols

Many studies reported the presence of the aliphatic compounds in* Chaenomeles* species. Nine phenols (**37–45**) were isolated from* C. speciosa*. Their structures of these compounds are shown in Table 5 and Figure 5.

### 2.6. Biphenyls

To date, only five biphenyls (**46–50**) were obtained from the ethanolic extract of* C. speciosa*. Their structures of these compounds are shown in Table 6 and Figure 6.

### 2.7. Others

In addition to the above-mentioned main components, other components (Table 7 and Figure 7) are also found in* Chaenomeles* species, such as fatty acid (**51–53**), quinic acids (**57**-**59**), coumarin (**61** and** 62**), and steroids (**63** and** 64**).

## 3. Pharmacological Activities

Various natural compounds have served huge industrial and individual demands in curing multiple diseases due to their potential pharmacological properties. This lust gained attention of the scientists to continue exploration of such similar plants and compounds bearing similar medicinal importance. Dried* Chaenomeles* fruits are being used as traditional herbal medicines since centuries within mainland of China to cure dysentery, prosopalgia, rheumatoid arthritis, cholera, beriberi, vitamin C deficiency syndrome, enteritis, and hepatitis [14, 30]. Presence of complex compounds such as phenolics, tannins, multiple organic acids, glycosides, and flavones in* Chaenomeles* made it an important plant having diverse pharmacological properties [31, 32]. The following sections will highlight and update the various pharmacological properties of* Chaenomeles* plant reported in recent years.* Chaenomeles* plant has said to possess diverse biological functions, due to which it has been in use since centuries in traditional Chinese medicine [13, 30, 33]. Modern day research also confirmed that* C. speciosa* is enriched in diverse pharmacological and biological properties particularly anti-inflammation, immunomodulation, antimicrobial, antitumor, and antioxidant actions are notable [33, 34].

### 3.1. Antioxidant Property

The botanical extracts are a potential source of natural antioxidants with almost no side effects [13]. The extracts of* C. speciosa* fruits have very strong antioxidant ability. In a recent study, it was claimed that* C. speciosa* have a strong potential for scavenging free radicals, i.e., various reactive oxygen species (ROS) and free nitrous oxide mainly due to the presence of strong antioxidant compound quercetin [17]. In atherosclerosis, level of LDL increased with decreased antioxidant capability in the blood due to the formation of LDL-oxidation complexes [35], which was successfully treated with the application of powdered* C. speciosa* that was thought to be due to its higher antioxidant nature of the plant parts that increased increasing the antioxidant levels in the blood and declining the cholesterol levels [36]. Moreover, various polysaccharides are being extracted from* C. speciosa* and reported to possess strong antioxidant activity [37].

A huge amount of flavonoid contents reported in* C. speciosa* has shown significant reduction in peroxide levels in lard, removed DPPH, and deoxidized the iron (Fe^3+^) in a concentration-dependent fashion, demonstrating its strong antioxidant nature compared to ascorbic acid (vitamin C) [38]. Moreover, quercetin and 3,4-dihydroxybenzoic acid which were extracted from* C. speciosa* showed higher inhibition for DPPH (2,2-diphenyl-1-picrylhydrazyl) and neuraminidase [17]. The antioxidants activity in five species of* Chaenomeles* (Mugua) was studied and about 44 fractions were prepared that showed a potent and stable free-radical DPPH and the hydroxyl radicals scavenging activity [39].

### 3.2. Anti-Inflammatory and Analgesic Activities

*C. speciosa* plant also being administered in inflammatory and immune related issues and glucosides present in the extracts have strong anti-inflammation as well as immunoregulatory properties [34, 40]. Methyl-3-hydroxybutanedioic ester found in* C. speciosa* has also been reported to possess strong anti-inflammatory effects particularly in inflammatory avian influenza and dyspepsia [17]. Various plant extracts even in fractions have also been reported to possess good potential against inflammations, pains and antianalgesic activity mainly due to the presence of chlorogenic acid [13]. Some of the* Chaenomeles* polysaccharides also showed anti-inflammatory activities [41]. In another study, it was reported that* Chaenomeles* fractions control the calcium channels, due to which it has got higher analgesic activity [42].* C. speciosa* plant is in use since long time to treat rheumatoid arthritis in various parts of China primarily due to its antinociceptive and anti-inflammatory potential [40]

### 3.3. Antiatherosclerotic Effects

As arteriosclerosis is a systemic condition, hyperlipidemias, especially oxidized low-density lipoprotein (LDL), are major elements that initiate this route and result in the formation of plaque [43]. Atherosclerosis is a condition, where the level of antioxidants decreased within the blood due to oxidation of LDL. Level of LDL increased with decreased antioxidant capability in the blood due to the formation of complexes [35], which was successfully treated by the application of powdered* C. speciosa*. It was thought to be due to the higher antioxidant nature of the plant parts by increasing the antioxidant levels in the blood and declining the cholesterol levels [36].

### 3.4. Antitumor and Immunomodulatory Activities

Cancer is the main cause of a large number of deaths across the world. Most of the cytotoxic drugs being used to cure cancer tissues have also been reported to be harmful to the normal tissues [44]. An average molecular weight water-soluble polysaccharide was successfully extracted from* C. speciosa* that is composed of glucose, rhamnose, galactose, and arabinose. This polysaccharide is good in inhibiting the tumor growth in the mice along with improvement in delayed type hypersensitivity and higher secretion of interleukin- (IL-) 2, TNF-*α*, and IFN-*γ* in blood serum [45]. It is further suggested that the antitumor effects of this polysaccharide might be due to the association with its potent immunostimulatory activity* in vivo* [45]. Many of the plant based polysaccharides are less nontoxic with hardly any serious problems compared with certain synthetic compounds and hence make these polysaccharides a superior choice for modern medication [46, 47].

It was in the mid-1970s, when it was said for the first time that various organic acids present in* C. speciosa* have strong antitumor activity studied on Ehrlich ascites carcinoma in mice [48], and this antitumor activity is because of the presence of multiple terpenoids [49, 50]. Among various acids present in this plant, there are betulinic, maslinic, oleanolic, and ursolic acids which are the prominent triterpenoid chemicals [51]. Oleanolic and ursolic acids in plants have inhibitory effects on estrogen receptor-negative breast cancer, osteosarcoma cells, and HuH7 human hepatocellular carcinoma cells, which induce quick apoptosis in the cancer cells [51–53]. Moreover, various acids present in the plant extracts including maslinic acids showed potential antiangiogenic properties on non-small-cell and lung cancer cells [51, 54].* In vivo*, ethanolic extracts of* C. speciosa* checked the growth of tumors along with increased immune responses in mice, while Foxp3, TGF-*β*, and PD-L1 protein expression levels were reduced within the tumors [3]. It was the pioneer study that explained ethanolic extracts of* C. speciosa* which strongly inhibited the growth and invasion of tumors by direct killing of cancerous cells as well as enhanced immune responses [3].

### 3.5. Antidiarrhea

Various organic acids (betulinic, oleanolic, and ursolic acids) are the active components in fruits of* C. speciosa* which are regarded as potent therapeutic candidates in treating LT-induced diarrhea [55]. Moreover, studies used the extracts of* C. speciosa* showing strong antimicrobial activity and analgesic effects [13, 33]. It is further suggested that G-protein-AC-cAMP regulated signaling has a major role in reducing the inflammation and deterioration of joints in arthritis rats [40]. Intervention of intracellular signaling cascades in synoviocytes by using glucosides of* C. speciosa* may consequently suppressed the deterioration of bones and reduction of inflammations in autoimmune rat models. The major and prominent effect of* C. speciosa * glucosides on higher cAMP levels in synoviocytes are thought to be linked with its inhibitory protein (Gi). Moreover, this Gi-protein mediated AC-cAMP signaling cascade is thought to be one of the vital procedures in reducing inflammations and modulating-immune responses by glucosides of* C. speciosa *[56–58].

### 3.6. *α*-Glucosidase Inhibitory

Among many varieties being cultivated within China,* C. speciosa* Var. Yunnan showed maximum antioxidant potential along with *α*-glucosidase inhibition [59]. Moreover, another study confirmed that, in* C. speciosa* total amount of polysaccharides, flavonoids, ursolic acid, and polyphenols are the prominent phytochemicals bearing good antioxidant properties. In addition to it, complete polysaccharides and flavonoid contents are mainly involved in inhibition of *α*-glucosidase activity of* C. speciosa* [60]. Such studies shall be helpful in qualitative evaluation of the* C. speciosa* and its implementation in different industries. Moreover, peels can be a good source of inhibitors of *α*-glucosidase activity and antioxidants within the broad range of pharmaceutical as well as related industries [1]. Until now, many phytochemicals having strong *α*-glucosidase inhibition potential are under use as an oral hypoglycemic medicines to check hyperglycemia [60]. Furthermore, exploration of *α*-glucosidase inhibitors is of supreme priority for finding novel antidiabetic drugs [61].

### 3.7. Antiviral Activity

The viral particles spread and replicates within the host cells cause severe disturbances and conditions. Recently, many influenza viral epidemics have been out broken in many parts of the world. The annual report by World Health Organization (WHO) published early in 2018 explained that data from about all over the world laboratories has confirmed many incidents of epidemic influenza H1N1 2009 that caused almost 13,554 mortalities [17]. The birds influenza virus mainly generated the oxidative stress and amplify the inflammations within the patient. Quercetin, 3,4-dihydroxybenzoic acid and methyl-3-hydroxybutanedioic esters, which were extracted from* C. speciosa*, pose a synergistic effect during the treatment of avian influenza and hence proved as prospective and strong antiviral compounds [17]. Avian influenza is frequent and followed with virus invasion along with increased oxidative stress and heavy inflammations. Furthermore, numerous effects of the* C. speciosa* compounds have a potential and specialized role in curing avian influenza; particularly quercetin would be a strong anti-inflammation and antiviral compound [17]. The oleanolic acid extracted from* C. speciosa *(20*μ*g/ml) robustly inhibits the replication of hepatitis B virus genome which showed a powerful inhibition ratio (29.33%) [62]. The* C. speciosa* plant extract contains powerful antioxidants in it that depicts it could potentially be used to cure new influenza A virus epidemics.

It is summarized that continuous research interests have explored plenty of natural compounds from* C. speciosa* plant. Such explorations of novel compounds from this plant have been said to possess strong medical importance. In certain disease aroused mainly of heavy oxidative stress, it is being replenished by the strong antioxidant nature of this plant. The flavonoids present in* C. speciosa *mainly reduce peroxide and LDL levels, remove DPPH and deoxidize free cellular iron (Fe^3+^), and hence pose a strong antioxidant effect. Moreover,* C. speciosa* plant also contributed its immune modulatory and anti-inflammatory effects by reducing the expression of interleukins and related inflammatory factors. The various phytochemicals particularly the polysaccharides present in* C. speciosa* possess *α*-glucosidase inhibition potential and hence reduce hypoglycemic conditions. Similarly, antimicrobial and analgesic effects of the* C. speciosa* compounds are mainly following G-protein-AC-cAMP regulated signaling cascade to reduce the inflammation and deterioration of joints in arthritis rats. In different virus associated pathologies such as avian influenza, quercetin, 3,4-dihydroxybenzoic acid, and methyl-3-hydroxybutanedioic esters reduce the oxidative stress and expression of many inflammatory factors caused by the viral pathogen and hence relive the pains and condition of the patient. Moreover, oleanolic acid present in* C. speciosa* efficiently inhibits the replication of hepatitis B virus genome.

## 4. Conclusions

It is concluded that* C. speciosa* have broad scope in industry and in medicines. Not only the leaves and fruits of* C. speciosa* plant, but various other parts which include roots, seeds, bark twigs, and flowers, all have long clinical trials history in curing many human ailments. However, the maximum accessible data concerning the chemical composition and their broad pharmacological properties of various parts of* C. speciosa* plants is quiet less that make it more appealing for in-depth investigations.

## Figures and Tables

**Table 1:** Triterpene compounds from *C. speciosa*.

No.	Compounds	Parts	Ref.
**1**	ursolic acid	fruits	[16–18]
**2**	3-*O*-acetyl ursolic acid	fruits	[16, 17, 19]
**3**	3-*O*-acetyl pomolic acid	fruits	[19, 20]
**4**	tormentic acid	fruits	[16, 17]
**5**	speciosaperoxide	fruits	[16, 17]
**6**	3*β-*acetoxyurs-11-en-13*β*,28-olide	fruits	[16, 17]
**7**	dihydrotomentosolic acid	twigs	[21]
**8**	ilexgenin B	twigs	[21]
**9**	betulinic acid	fruits, twigs	[19–21]
**10**	23-hydroxybetulinic acid	twigs	[21]
**11**	pycarenic acid	twigs	[21]
**12**	oleanolic acid	fruits	[15–18, 20, 22]
**13**	maslinic acid	fruits, twigs	[16, 17, 21]

**Table 2:** Sesquiterpenoid compounds from *C. speciosa*.

No.	Compounds	Parts	Ref.
**14**	speciosaoside A	fruits	[23]
**15**	(1'*R*,3'*R*,5'*R*,8'*S*)-epi-dihydrophaseic acid-*β*-D-glucoside	fruits	[23]
**16**	Vomifoliol	fruits	[16, 17]
**17**	roseoside	fruits	[16, 17, 24]
**18**	(6*S*,7*E*,9*R*)-6,9-dihydroxy-4,7-megastigmadien-3-one 9-*O*-[*β*-D-xylopyranosyl(1→6)-glucopyranoside]	fruits	[16, 17, 24]

**Table 3:** Flavonoid compounds from *C. speciosa*.

No.	Compounds	Parts	Ref.
**19**	quercetin	fruits	[17, 25]
**20**	catechin	fruits	[18]
**21**	(-)-epicatichin	fruits	[26]
**22**	specpolyphenol A	fruits	[27]
**23**	pelargonidol chloride	petals	[28]
**24**	pelargonidin 3-*O*-*β*-D-glucopyranoside	petals	[28]
**25**	pelargonidin-3-galactoside	petals	[28]
**26**	cyanidin 3-*β*-*O*-glucoside	petals	[28]
**27**	cyanidin 3-*O*-*β*-galactopyranoside	petals	[28]

**Table 4:** Phenylpropanoid compounds from *C. speciosa*.

No.	Compounds	Parts	Ref.
**28**	cinnamic acid	fruits	[26]
**29**	caffeic acid	fruits	[22, 26]
**30**	n-butyl caffeate	fruits	[22]
**31**	1-*O*-p-coumaroyl-*β*-D-glucose	fruits	[24]
**32**	specphenoside A	fruits	[27]
**33**	chlorogenic acid	fruits	[26]
**34**	methyl chlorogenate	fruits	[26]
**35**	ethyl chlorogenate	fruits	[20]
**36**	5-*O*-caffeoylquinic acid butyl ester	fruits	[26]

**Table 5:** Phenolic compounds from *C. speciosa*.

No.	Compounds	Parts	Ref.
**37**	hydroquinone	fruits	[25]
**38**	4-hydroxybenzoic acid	twigs	[21]
**39**	3,4-dihydroxybenzoic acid	fruits	[17, 20, 25, 26]
**40**	protocatechuic acid ethyl ester	fruits	[26]
**41**	vanilloloside	fruits	[27]
**42**	di-*O*-methylcrenatin	fruits	[27]
**43**	3,5-dihydroxyphenethyl alcohol 3-*O*-*β*-glucopyranoside	fruits	[27]
**44**	1,2,4-hydroxybenzene	fruits	[24]
**45**	gallic acid	fruits	[20, 24]

**Table 6:** Biphenyls from *C. speciosa*.

No.	Compounds	Parts	Ref.
**46**	2',4'-dimethoxyaucuparin	twigs	[21]
**47**	aucuparin	twigs	[21]
**48**	2'-methoxyaucuparin	twigs	[21]
**49**	chaenomin B	twigs	[21]
**50**	chaenomin A	twigs	[21]

**Table 7:** Other compounds from *C. speciosa*.

No.	Compounds	Parts	Ref.
**51**	3-methyl-3-hydroxylbutanedioic ester	fruits	[17, 25, 29]
**52**	triacontanoic acid	fruits	[26]
**53**	nonacosan-10-ol	fruits	[15, 18]
**54**	5-hydroxyme thyl-furan-2-carbaldehyde	fruits	[26]
**55**	5-hydroxynicotinic acid	fruits	[24]
**56**	shikimic acid	fruits	[18]
**57**	(-)-quinide	fruits	[24]
**58**	quinic acid	fruits	[18]
**59**	quinic acid butyl ester	fruits	[26]
**60**	kojic acid	fruits	[20]
**61**	esculetin	fruits	[26]
**62**	7,8-dihydroxycoumarin	fruits	[26]
**63**	*β*-sitosterol	fruits	[15, 18, 22]
**64**	daucosterol	fruits	[15, 18, 22]
